# The ethanol extract of *Garcinia subelliptica* Merr. induces autophagy

**DOI:** 10.1186/s12906-021-03454-4

**Published:** 2021-11-10

**Authors:** Kyun Ha Kim, Ji Yeon Lee, Wan Yi Li, Sangwoo Lee, Han-Sol Jeong, Jun-Yong Choi, Myungsoo Joo

**Affiliations:** 1 School of Korean Medicine, Pusan National University, Yangsan, 50612 Republic of Korea; 2 Institute of Medicinal Plants, Yunnan Academy of Agricultural Sciences, Kunming, Yunnan 650224 China; 3 International Biological Material Research Center, Korea Research Institute of Bioscience and Biotechnology, Daejeon, 34141 Republic of Korea; 4 Lung Cancer Clinic, Pulmonary Medicine Center, Korean Medicine Hospital, Pusan National University, Yangsan, 50612 Republic of Korea

**Keywords:** *Garcinia subelliptica* Merr., Human lung carcinoma cells, mTOR, AMPK, Autophagy

## Abstract

**Background:**

*Garcinia subelliptica* Merr. is a multipurpose coastal tree, the potential medicinal effects of which have been studied, including cancer suppression. Here, we present evidence that the ethanol extract of *G. subelliptica* Merr. (eGSM) induces autophagy in human lung adenocarcinoma cells.

**Methods:**

Two different human lung adenocarcinoma cell lines, A549 and SNU2292, were treated with varying amounts of eGSM. Cytotoxicity elicited by eGSM was assessed by MTT assay and PARP degradation. Autophagy in A549 and SNU2292 was determined by western blotting for AMPK, mTOR, ULK1, and LC3. Genetic deletion of AMPKα in HEK293 cells was carried out by CRISPR.

**Results:**

eGSM elicited cytotoxicity, but not apoptosis, in A549 and SNU2292 cells. eGSM increased LC3-II production in both A549 and, more extensively, SNU2292, suggesting that eGSM induces autophagy. In A549, eGSM activated AMPK, an essential autophagy activator, but not suppressed mTOR, an autophagy blocker, suggesting that eGSM induces autophagy by primarily activating the AMPK pathway in A549. By contrast, eGSM suppressed mTOR activity without activating AMPK in SNU2292, suggesting that eGSM induces autophagy by mainly suppressing mTOR in SNU2292. In HEK293 cells lacking AMPKα expression, eGSM increased LC3-II production, confirming that the autophagy induced by eGSM can occur without the AMPK pathway.

**Conclusion:**

Our findings suggest that eGSM induces autophagy by activating AMPK or suppressing mTOR pathways, depending on cell types.

**Supplementary Information:**

The online version contains supplementary material available at 10.1186/s12906-021-03454-4.

## Introduction

*Garcinia subelliptica* Merr. is a coastal tree species found in Japan, China, Taiwan, India, Sri Lanka, and the Philippines [1]. *G. subelliptica* Merr. has been known to contain various chemical constituents regulating bacterial infection, inflammation, and cancer [2]. For example, garcinielliptones were reported to inhibit the release of β-glucuronidase and lysozyme [3]. They also suppress superoxide formation from activated neutrophils and peripheral mast cells [4]. Xanthone isolated from the plant is cytotoxic to cancer cells [5], and benzophenonoids exhibit cytotoxicity to A549 non-small lung carcinoma cell, DU145 prostate carcinoma cell, and KB nasopharyngeal carcinoma cells [6]. These results suggest that the potential pharmacological significance of the plant is high. However, the use of plant extract as an herbal remedy appears relatively limited. A recent report showed that the ethanol extract of the leaf of *G. subelliptica* has anti-inflammatory activity [7]. The leaf extract reduced nitric oxide production, cyclooxygenase-2, and proinflammatory cytokines in RAW264.7 cells stimulated with lipopolysaccharide. These results are in accord with the reports that the plant contains numerous constituents contributing to anti-inflammatory effects. Given the cytotoxicity of some constituents of the plant to several cancer cells, it would be worth exploring the plant extract as a potential herbal remedy to treat cancer.

Cytotoxicity could trigger autophagy, a cellular system that enables cells to cope with a constantly changing environment [8]. Autophagy is now considered a process of homeostasis that involves the digestion of self-proteins and organelles, by which cells can deal with various environmental challenges such as starvation [9] and infectious and other diseases [10]. Detailed molecular processes for autophagy are well-documented [11]. Mechanistically, autophagy is primarily regulated by two essential kinases, the mechanistic target of rapamycin (mTOR) and the AMP-activated protein kinase (AMPK). In a metabolically favorable environment, mTOR becomes phosphorylated at Ser2448 and active [12]. The activated mTOR senses energy-rich environmental cues, prompting anabolism and suppressing autophagy by phosphorylating ULK1 at S757 [13]. On the other hand, in a metabolically adverse environment where glucose or amino acids are limited and catabolism is required for cell survival [14], AMPK becomes activated and increases autophagy while suppressing mTOR activity, resulting in enhanced catabolism [15]. When sensing the lack of ATP, AMPK phosphorylates several serine residues in ULK1 [16], including S317 and S777 [17]. ULK1 phosphorylated at these sites triggers autophagosome formation, making autophagy start [14]. Concurrently, AMPK suppresses mTOR activity to stop anabolism. For suppressing mTOR activity, AMPK phosphorylates and activates TSC2, an mTOR upstream regulator, while phosphorylating and inactivating RAPTOR, a subunit of mTORC1 [13]. Phosphorylating both TSC2 and RAPTOR contributes to the decrease of mTOR activity. Reduced mTOR activity results in decreased phosphorylation of ULK1 at S757 [17]. Phosphorylation at S757 in ULK1 is known as inactivating phosphorylation because it blocks autophagosome formation [17]. Once ULK1 becomes active, as indicated by phosphorylation at S317 and but not at S757, autophagy process initiates to form autophagosomes [18]. During autophagosome formation, microtubule-associated protein 1A/1B-light chain 3 (LC3) in the cytosol is truncated to LC3-I and subsequently conjugated with phosphatidylethanolamine to form LC3-II [19]. Since LC3-II is mainly found in autophagosomes, LC3-II serves as a critical biomarker for autophagy formation [20].

Given the cytotoxicity exerted by several constituents of *G. subelliptica* Merr., we set out to explore the possible usage of the plant extract as herbal medicine to treat cancer. In this study, we show that the leaf ethanol extract of *G. subelliptica* Merr. (eGSM) exhibited cytotoxicity to two different lung cancer cells, A549 and SNU2292. The cytotoxicity by eGSM appeared to be related to autophagy but not apoptosis. Furthermore, we present evidence that eGSM induced autophagy by activating AMPK and suppressing mTOR activity. Our results provide additional ethnopharmacological significance of *G. subelliptica* Merr., which could be a scientific basis for developing a possible herbal remedy using *G. subelliptica* Merr. extract.

## Materials and methods

### Reagents and antibodies

The ethanol extract of the leaf of *Garcinia subelliptica* Merr. (catalog # FBM124-035) was purchased from the Korea Research Institute of Bioscience and Biotechnology (Daejeon, Korea). Doxorubicin (D1515), E-64 (E8640), pepstatin A (P4265), and hydroxychloroquine (H0915) were purchased from Sigma-Aldrich (St. Louis, MO, USA), and rapamycin (#13346) was from Cayman (Ann Arbor, MI, USA). Antibodies against PARP (#9532), mTOR (#2972), phosphor-mTOR (S2448, #2971), ULK1 (#8054), phospho-ULK1(S757, #6888), and phospho-ULK (S317, #12753) were procured from Cell signaling (Danvers, MA, USA). Anti-p62/SQSTM1 antibody was obtained from Abcam (Cambridge, UK). Antibodies against β-actin (sc-477,778) and AMPKα (sc-25,792) were obtained from Santa Cruz Biotechnology (Santa Cruz, CA, USA), and LC3 (L7543) was from Sigma-Aldrich.

### Assessment of cytotoxicity

Cytotoxicity was determined using a Vybrant® MTT assay kit (Thermo Fisher Scientific, Waltham, MA, USA). Cell culture plates of A549 and SNU2292 were treated with various amounts of eGSM dissolved in PBS for 16 h and measured by a plate reader (BioTeK, VT, USA), as instructed by and the manufacturer. The percentage of live cells was calculated over untreated cells. The assay was conducted in triplicate and repeated three times.

### Cells

A549 and HEK293 cells were purchased from American Type Culture Collection (Rockville, MD, USA) and SNU2292 cells [21] from Korean Cell Line Bank (Seoul, Korea). Cells were cultured in Dulbecco’s Modified Eagle’s Medium (DMEM) containing L-glutamine (200 mg/L), 10% (v/v) heat-inactivated fetal bovine serum (FBS), and 100 U/ml penicillin and 100 μg/ml streptomycin in a humidified incubator at 37 °C and 5% CO_2_.

### Genetic deletion of AMPKα by CRISPR

AMPKα1/2 genes were genetically ablated by using the CRISPR-Cas9 system. The guide sequences were designed using the CRISPR design tool (http://www.rgenome.net/cas-designer/). Guide sequences for AMPKα1 were as follows: 5′-GCGAGCTTCGTCCTCA TGCAGGG-3′ and 5′-TACTCAATCGACAGAAGATT-3′. Those for AMPKα2 were 5′-GAAGATCGGACACTACGTGC-3′ and 5′-CTACGTGCTGGGCGACACGC-3′. The annealed AMPKα1 or α2 guide sequences were inserted into the pX459 vector (plasmid No. 62988, Addgene, Watertown, MA, USA). AMPKα1 and AMPKα2 guide sequences were transfected into the HEK293A cells (Thermo Scientific). After the transfected cells were treated with puromycin (1 μg/ml) for 3 days, the HEK293A cell was individually seeded into 96 well plates by BD FACS Aria™ III sorter (BD, San Jose, CA, USA). A clone that lacks expression of AMPKα protein was screened by western blot.

### Western blot analysis

Total proteins were prepared by Pierce™ IP lysis buffer (Thermo Fisher Scientific), the amount of which was determined by Bradford (Bio-Rad, Hercules, CA, USA). Equal amounts of proteins loaded onto each lane were separated on NuPAGE gel (Thermo Fisher Scientific) and blotted to PVDF membrane (Bio-Rad). After blocked with 5% non-fat dry milk, the membrane was incubated with appropriate primary antibodies and then with HRP-conjugated secondary antibodies. Proteins of interest were revealed by using SuperSignal®West Femto (Thermo Fisher Scientific). Membranes were stripped with Restore™ Western Blot Stripping Buffer per the manufacturer’s protocol (Thermo Fisher Scientific).

### Statistical analysis

One-way analysis of variance (ANOVA) tests was used, along with Tukey’s post hoc test (InStat, Graphpad Software, Inc., San Diego, CA). Data are shown in the mean ± SEM (Std. Error) of three measurements. *P* (≥0.05) was considered statistically significant.

## Results

### Cytotoxicity of eGSM

**Fig. 1:**
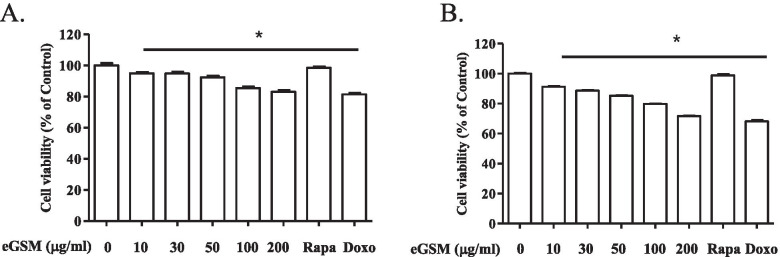
Cytotoxicity of eGSM. Human lung adenocarcinoma A549 (**A**) or SNU2292 (**B**) was treated with indicated amounts of eGSM for 16 h. Cytotoxicity was determined by MTT assay. Doxorubicin (2 μg/ml) was included as a positive control for cytotoxicity. Rapamycin (10 μM), the inhibitor of mTOR, was also tested for its cytotoxicity. The experiment was performed three times, and the representative results are shown here. Data are shown in the mean ± SEM of three measurements. *P was less than 0.05, compared to untreated controls

Since some of the chemical constituents in GSM were reported to show anti-cancer effects, including cytotoxicity and apoptosis of cancer cells [2], we hypothesized that GSM has similar activity. To test our hypothesis, we used the ethanol extract of GSM leaves (eGSM) and tested whether eGSM has a cytotoxic effect using lung carcinoma cells. One million A549 cells were treated with increasing amounts of eGSM for 16 h, and then the cytotoxicity elicited by eGSM was determined by MTT assay. As shown in Fig. 1A, eGSM decreased the cell viability as low as 10 μg/ml in a statistically significant fashion. The viability of A549 cells was further reduced as the amount of eGSM increased; the cytotoxicity at 200 μg/ml was comparable to doxorubicin (2 μg/ml). Similar effects of eGSM were observed in SNU2292 cells (Fig. 1B). As in A549, eGSM decreased the viability of SNU2292 as the amount of eGSM increased. Together, these results suggest that eGSM has a cytotoxic effect on the two different lung carcinoma cell lines.

### Cytotoxicity of eGSM is associated with autophagy

**Fig. 2:**
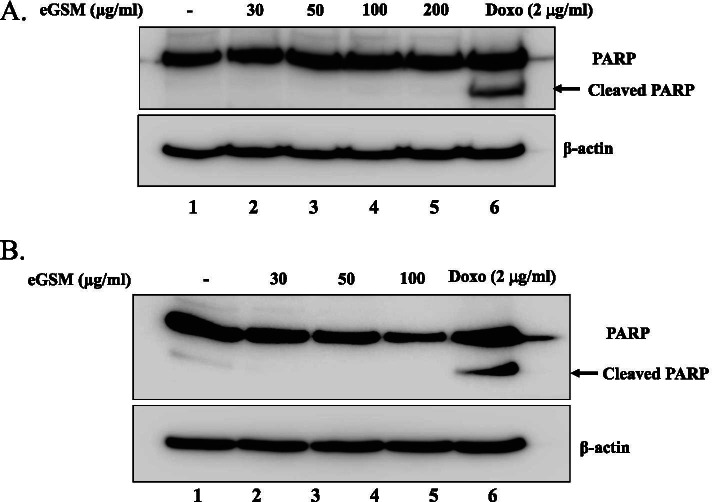
eGSM did not induce apoptosis. **A** A549 cells were treated with indicated amounts of eGSM for 16 h (lanes 2 to 5). Total proteins were extracted and measured by Western blotting for PARP. Cleaved PARP produced during apoptosis was indicated by an arrow. Similar experiments were performed with SNA2292 cells (**B**). The membranes blotted for PARP were stripped and blotted with an anti-β actin antibody for equal loading of samples

The possibility that apoptosis is responsible for the cytotoxicity of eGSM was tested by measuring the proteolytic cleavage of poly (ADP-ribose) polymerase (PARP), which occurs by caspases activated during apoptosis [22]. A549 cells were treated with increasing amounts of eGSM. At 16 h after treatment, total proteins were extracted and analyzed by Western blotting for PARP. As shown in Fig. 2A, while doxorubicin, a cancer drug that promotes apoptosis, generated a cleaved PARP fragment (arrow), eGSM did not cleave PARP in A549 cells. A similar experiment was performed with SNU2292 cells (Fig. 2B). As in A549, while doxorubicin triggered apoptosis, as evidenced by the cleaved PARP, eGSM failed to do it in SNU2292 cells. These results suggest that the cytotoxicity by eGSM is not associated with apoptosis of the cancer cells.

**Fig. 3:**
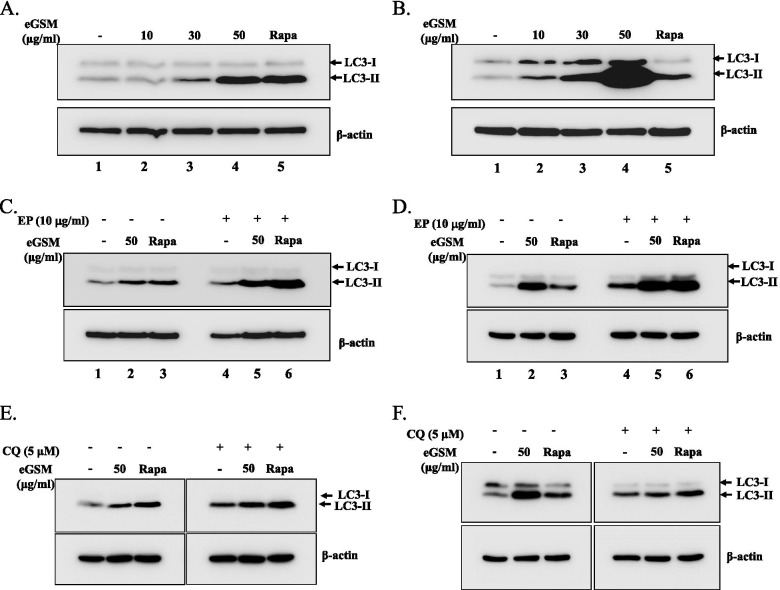
eGSM induced autophagy. A549 cells (**A**) or SNU2292 cells (**B**) were treated with indicated amounts of eGSM for 16 h (lanes 2 to 4), along with rapamycin (10 μM, 6 h). A549 (**C**) or SNU2292 (**D**) was treated with 10 μg of the mixture of E-64 and pepstatin A (EP) (1:1 ratio) 3 h prior to eGSM treatment as indicated (lanes 4 to 6). Additionally, A549 (**E**) or SNU2292 (**F**) was treated with 5 μM of hydroxychloroquine for 3 h before eGSM treatment. Total proteins were extracted, fractionated, and blotted for LC3 with anti-LC3 antibody. Two modified forms of LC3 proteins, LC3-I and LC3-II, are indicated by arrows. Blotted membranes were stripped and probed with anti-β actin antibody

Since no apoptosis seemed involved in the cytotoxicity of eGSM, we then tested the possibility that eGSM induces autophagy known to be closely associated with cancer [23]. To determine autophagy, we analyzed the level of LC3-II protein, a well-characterized autophagy marker found in autophagosomes [20]. A549 cells were treated with different amounts of eGSM for 16 h; total proteins were extracted and analyzed by Western blotting for LC3 proteins. As shown in Fig. 3A, eGSM treatment increased the level of LC3-II in A549 cells. Similarly, eGSM increased the production of LC3-II in SNU2292, the level of which appeared to be much higher than A549 (lane 4 in Fig. 3A and B). In a parallel experiment, eGSM decreased the level of p62/SQSTM1 (Supplement Fig. 1). Given the level of p62 tends to be diminished because of incorporation into autophagosome and degradation during autophagy [24], these results suggest eGSM induces autophagy. To confirm that eGSM inducing LC3-II is related to autophagy, we examined the transit nature of autophagy by blocking the autophagy flux [25]. Cells were treated as above and treated with E-64 (5 μM) and pepstatin A (5 μM) 3 h before cell harvest. As shown in Fig. 3C, the level of LC3-II induced by eGSM (lane 2) was further increased by treating these protease inhibitors (lane 5). In SNU2292 (Fig. 3D), which showed a robust LC3-II expression upon eGSM treatment, the protease inhibitors failed to increase the expression of LC3-II by eGSM (lane 2) (lane 5). To further confirm these observations, we treated cells with hydroxychloroquine (5 μM) that blocks the fusion between autophagosomes and lysosomes [26]. As shown in Fig. 3E and F, the results with chloroquine were consistent with those in Fig. 3C and D. It would be possible if autophagy in SNU2292 occurs robustly and rapidly upon eGSM treatment, which outpaces degradation of LC3-II. Regardless of detailed mechanisms, these results strongly suggest that eGSM induces autophagy in A549 and SNU2292 cells.

### eGSM induces autophagy in A549 and SNU2292 in divergent pathways

**Fig. 4:**
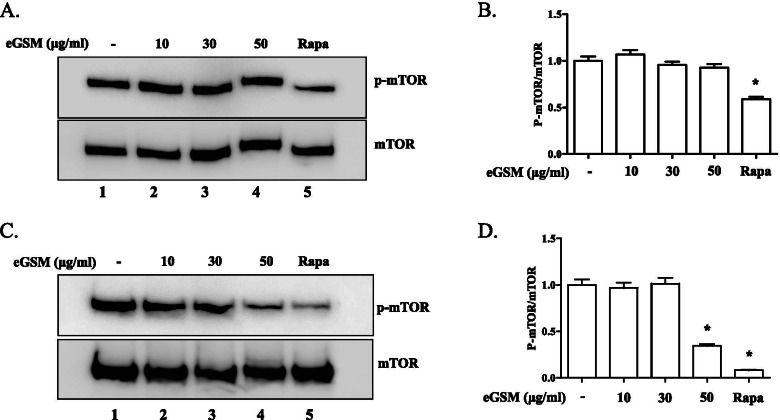
eGSM decreased the phosphorylated mTOR in SNU2292, not in A549. A549 cells (**A**) or SNU2292 cells (**C**) were treated with indicated amounts of eGSM for 16 h (lanes 2 to 5), along with rapamycin (10 μM, 6 h). Total proteins were extracted and measured by Western blotting for mTOR phosphorylated at S2448. The blotted membrane was stripped and reprobed for mTOR to ensure equal loading. The bands were analyzed by ImageJ and the relative levels of the phosphorylated mTOR over mTOR in A549 (**B**) and SNU2292 (**D**) are shown. Three measurements of each band are presented as in the mean ± SEM. *P was less than 0.05, compared to untreated controls

**Fig. 5:**
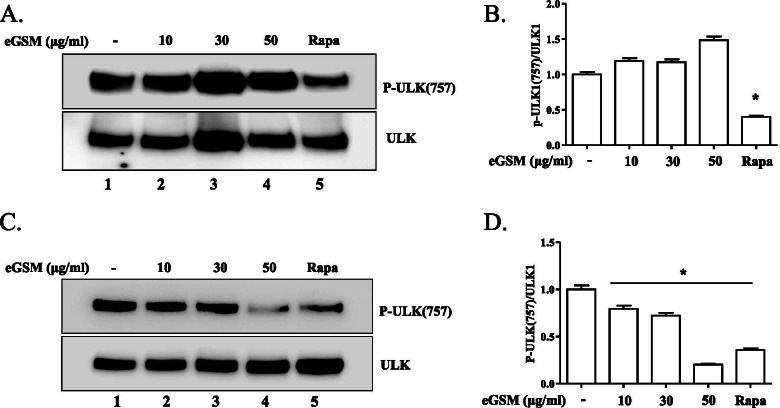
eGSM decreased mTOR activity in SNU2292, not in A549. From A549 cells (**A**) or SNU2292 cells (**C**) treated with indicated amounts of eGSM for 16 h (lanes 2 to 4), total proteins were extracted and analyzed for ULK1 phosphorylated at S757, a target of mTOR kinase activity. Rapamycin (10 μM, 6 h) was included as a negative regulator of mTOR kinase activity (lane 5). The blotted membrane was stripped and reprobed for ULK1 to ensure equal loading. The bands were analyzed by ImageJ, and the relative levels of the phosphorylated ULK1 over ULK1 in A549 (**B**) and SNU2292 (**D**) are shown, where each band was measured three times and shown as in the mean ± SEM. *P was less than 0.05, compared to untreated controls

Since autophagy occurs when mTOR is suppressed or AMPK is activated [13], we first tested whether eGSM inducing autophagy in lung carcinoma cells is associated with suppressing mTOR. A549 cells were treated with different amounts of eGSM, and the phosphorylation of mTOR at S2448, indicative of activated mTOR, was examined by Western blotting (Fig. 4A). Unlike rapamycin, an inhibitor of mTOR, that decreases the phosphorylation at S2448 (lane 5 in Fig. 4A and 5^th^ column in Fig. 4B), eGSM did not significantly suppress the phosphorylation at the S2448 of mTOR (Fig. 4B), suggesting that eGSM inducing autophagy in A549 is unrelated to the suppression of mTOR. However, when similar experiments were performed with SNU2292 cells (Fig. 4C), eGSM significantly suppressed the phosphorylation at the S2448 of mTOR (4th column in Fig. 4D), suggesting that, unlike A549, eGSM inducing autophagy in SNU2292 is associated with suppressing mTOR activity. To verify the differential suppression of mTOR by eGSM, we examined the phosphorylation of ULK1 at S757, a serine residue targeted by active mTOR. Consistent with the results in Fig. 4, while eGSM not affecting the phosphorylation of ULK1 at S757 in A549 cells (Fig. 5A and B), eGSM suppressed the phosphorylation of ULK1 at S757 in SNU2292 (Fig. 5C and D). Together, these results suggest that eGSM inducing the autophagy of SNU2292 but not of A549 cells is associated with the suppression of mTOR activity.

**Fig. 6:**
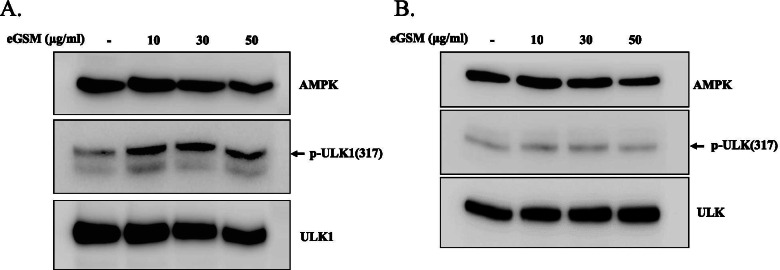
eGSM increased AMPK activity in A549 but not in SNU2292. **A** A549 cells were treated with indicated amounts of eGSM for 16 h. Total proteins were extracted and analyzed for ULK1 phosphorylated at S317, a target of AMPK kinase activity. The blot of ULK1 phosphorylated at S317 was stripped and reblotted for ULK1 to ensure equal loading. **B** SNU2292 cells were treated with eGSM, as in (**A**). ULK1 and phosphorylated ULK1 at S317 were similarly examined

Since eGSM inducing autophagy in A549 appeared not associated with mTOR, we tested whether eGSM activates AMPK instead for autophagy. A549 cells were treated with different amounts of eGSM, and total proteins were extracted and analyzed by Western blotting for the phosphorylation of ULK1 at S317, a serine residue targeted by AMPK to prompt autophagy. As shown in Fig. 6A, eGSM induced the phosphorylation at the S317 of ULK1, suggesting that eGSM activates AMPK. In similar experiments with SNU2292, eGSM failed to phosphorylate it at S317 (Fig. 6B). Together, these results indicate that eGSM activates AMPK to induce autophagy in SNU2292 but not in A549 cells.

### eGSM induces autophagy without AMPK

**Fig. 7:**
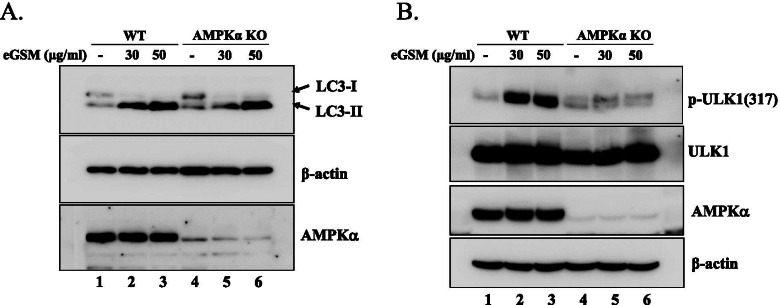
eGSM induced autophagy in the absence of AMPK activity. **A** The expression of AMPKα1/2 genes was inactivated by CRISPR in HEK293 cells (lanes 4 to 6, bottom panel). Along with WT (lanes 1 to 3), AMPKα KO cells were treated with two different amounts of eGSM for 16 h. Total proteins were isolated and analyzed by western blotting for LC3-1 and LC3-II (top panel), the membrane of which was stripped and reblotted for β-actin (middle panel). **B** WT (lanes 1 to 3) and AMPKα KO cells (lanes 4 to 6) were treated as in (**A**), and ULK1 phosphorylated at S317 (top panel), a serine phosphorylated by activated AMPK, and ULK1 (2nd panel) and were analyzed by Western blot. AMPKα and β-actin were similarly analyzed with stripped membranes

Our findings that eGSM suppressed mTOR in SNU2292 while activating AMPK in A549 for autophagy induction suggest that eGSM inducing autophagy is achievable by either suppressing the mTOR or activating the AMPK pathway. To test this possibility, we genetically nulled the expression of AMPKα by CRISPR in HEK293 cells and tested whether eGSM induces the autophagy of this AMPKα knockout (KO) cell. As shown in Fig. 7A, eGSM produced LC3-II in both wild type (WT) and AMPKα KO cells, suggesting that, similar to the two lung adenocarcinoma cells, eGSM induces autophagy in HEK293 cells and eGSM induces autophagy even in the absence of AMPKα (lanes 4 to 6). To verify these observations, we further determined the lack of AMPK activity in AMPKα KO cells (Fig. 7B). WT or AMPKα KO cells were treated with eGSM, and total cell extracts were analyzed by Western blotting of ULK1 phosphorylated at S317, a serine residue targeted by active AMPK. As in A549 cells, eGSM induced the phosphorylation at the S317 of ULK1 in WT cells (lanes 2 and 3), suggesting that eGSM activates AMPK in HEK293 cells. In AMPKα KO cells, where AMPK activity was expected to be none, eGSM failed to phosphorylate ULK1 at S317, suggesting no induction of AMPK activity by eGSM in AMPKα KO cells (lanes 5 and 6). Combined with the results showing the production of LC3-II in AMPKα KO cells after eGSM treatment, these results suggest that eGSM induces autophagy without AMPK activity.

## Discussion

This study shows that the ethanol extract of GSM (eGSM) can induce autophagy in adenocarcinoma lung cancer cells A549 and SNU2292. eGSM treatment induced a robust production of LC3-II in both A549 and SNU2292, the accumulation of which was further enhanced by treatment of lysosomal proteases inhibitors, suggesting that eGSM induces autophagy. Induction of autophagy appeared to be caused by eGSM activating AMPK in A549 cells or eGSM suppressing mTOR in SNU2292 cells. Furthermore, we show that eGSM could induce autophagy in the absence of AMPK activity, suggesting that suppressing mTOR is sufficient for eGSM to induce autophagy. Together, our findings suggest that eGSM can trigger autophagy by either activating AMPK or suppressing mTOR, depending on cell type.

At the outset, we explored a possible anti-cancer effect of the ethanol extract of GSM (eGSM) using A549 and SNU2292. Our initial goal was set on the publications showing that some of the chemical constituents of GSM have a possible anti-tumor effect [5, 6, 27]. In those studies, potential anti-cancer effects of chemicals were proposed mostly based on cytotoxicity measured by MTT assays [5, 27, 28]. Consistent with these reports, our MTT assays showed that eGSM exhibited cytotoxicity in A549 and SNA2292 cells. However, given our results that eGSM induced autophagy, we would like to point out a caveat when interpreting cytotoxicity measured by MTT assays. It is well-documented that during autophagy, mitochondria population decreases, as mitochondria are wrapped in autophagosomes and digested after fusion with lysosomes [15]. As a result, oxidoreductase activity in a cell is likely decreased during autophagy, which leads to MTT poorly reduced [29]. Since a low level of formazan, a reduced form of MTT, is routinely interpreted as senescence or dying of cells, a high degree of autophagy induced by eGSM could be construed as high cytotoxicity and thus an anti-cancer effect.

Rapamycin treatment can result in cell death in certain cell types [30], suggesting that inhibiting mTOR activity alone can be sufficient for apoptosis. However, the concentration of rapamycin used in the study was only enough to induce autophagy but not cytotoxicity in both lung cancer cell lines, while eGSM induced both autophagy and cytotoxicity but not apoptosis. Given this result, eGSM inducing cytotoxicity to the cancer cells may be due to unknown mechanisms other than autophagy, per se, and apoptosis. We observed that inhibiting lysosomal proteases by E64/pepstatin A was less effective in accumulating LC3-II in eGSM-treated cells than in rapamycin-treated ones (Fig. 3C and D). This could happen if eGSM induced autophagosome formation robustly and abundantly so that autophagosome newly formed outnumbered lysosomes or lysosomal activities. Alternatively, if eGSM could slow down the fusion between autophagosome and lysosome, it is possible that the level of LC3-II would be steady, not substantially affected by lysosome inhibitors, although we don’t have evidence to back these possibilities. Regardless of the mechanisms, it is conceivable that eGSM generated autophagosomes unresolved by lysosomes within a cell, which could be toxic to the cell. In support of our hypothesis, there is a report showing that a high level of autophagosome within the cytoplasm is toxic to the cell [31]. This finding could help explain why eGSM showed cytotoxicity to lung cancer cells, along with autophagy.

There are several ways to measure autophagy, one of which is to detect LC3-II proteins. Since LC3-II is a well-characterized marker for autophagy, we chose to measure LC3-II to determine whether autophagy occurred. Our data show that eGSM increased LC3-II level in two different cancer cell lines, suggesting eGSM inducing autophagy. To understand how eGSM induces autophagy, we examined whether eGSM activates AMPK or suppresses mTOR. Since activated AMPK phosphorylates ULK1 at S317, triggering autophagosome formation [17], we tested whether eGSM phosphorylates ULK1 at S317. On the other hand, since active mTOR phosphorylates ULK1 at S757, blocking autophagosome formation [17], we examined whether eGSM decreases the phosphorylation at the S757 of ULK1. Our results show that eGSM increased the phosphorylation of ULK1 at S317 in SNU2292, suggesting that eGSM activates AMPK to promote autophagy. However, in A549 cells, eGSM suppressed the phosphorylation of ULK1 at S757, suggesting that eGSM suppresses mTOR instead in A549 cells. These results suggest that eGSM activates AMPK or suppresses mTOR, depending on cell type. Nevertheless, together with increased LC3-II, these results clearly show that eGSM induced autophagy in the two human adenocarcinoma lung cancer cell lines.

Intriguing results observed in this study are that, depending on cell types, eGSM appeared to use either AMPK or mTOR pathways to induce autophagy. This finding suggests that either activating AMPK or inactivating mTOR is sufficient for inducing autophagy. The data in Fig. 7, where a lack of AMPK activity did not deter autophagy caused by eGSM, also supported the notion that eGSM induced autophagy even in the absence of active AMPK. To our knowledge, there is no definite study about whether or not AMPK is requisite for autophagy, and our results show that autophagy can take place without AMPK activity involved.

**Fig. 8:**
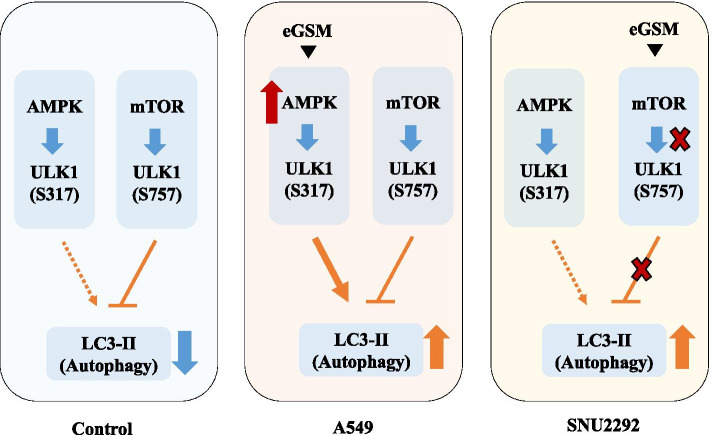
Schematics for eGSM to induce autophagy. Either activating AMPK or suppressing mTOR can induce autophagy. eGSM induced autophagy in A549 and SNU2298. Interestingly, eGSM activated AMPK while not affecting mTOR activity in A549 cells. By contrast, eGSM suppressed mTOR while not affecting AMPK activity in SNU2292 cells. How the two cells respond differently to eGSM warrants further studies. Given that eGSM triggered the both pathways resulting in autophagy, our results suggest that eGSM has a capability of inducing autophagy by activating AMPK activity and suppressing mTOR activity

It is clear that the mTOR pathway in both A549 and SNU2292 was intact. As shown in Fig. 4, mTOR in both cell types was phosphorylated at S2248, indicative of active mTOR [12]. In line with this, ULK1 was phosphorylated at S757, a serine residue targeted by active mTOR, as shown in Fig. 5. Additionally, rapamycin suppressed mTOR activity in both cell lines, suggesting that mTOR in both cell lines behaves as expected. Despite intact mTOR, however, eGSM failed to suppress mTOR in A549 while successfully suppressing mTOR in SNU2292. It remains unknown how the two different cancer cells chose different pathways for inducing autophagy when treated with eGSM. It is conceivable that eGSM could be metabolized differently in the two cancer cells. For instance, A549 cell metabolized eGSM to generate a metabolite that could activate AMPK but not the metabolite that suppresses mTOR activity (Fig. 8). Conversely, SNU2292 metabolized eGSM differently so that a metabolite generated could suppress mTOR, increasing autophagy, but no metabolite was available that would activate AMPK in SNU2292 cells. Based on these results, we could speculate that the two cancer cells have developed different enzymatic make-ups during the divergent cancer process, making cells respond differently to eGSM.

In this study, we show that eGSM induced autophagy in two different lung adenocarcinoma cells, A549 and SNU2292. eGSM activated the AMPK pathway for autophagy of A549, but suppressed mTOR for autophagy of SNU2292. In the absence of AMPK activity, eGSM induced autophagy. Together, our results suggest that eGSM can induce autophagy by activating AMPK or suppressing mTOR.

## Supplementary Information

**Additional file 1: Supplement Fig. 1.** A549 (A) or SNU2292 (B) cells were treated with indicated amounts of eGSM for 16 h. Total proteins were extracted, fractionated, and analyzed by western blot with α-p62 antibody. The membranes were stripped and reblotted with α-β-actin antibody. **Supplement Fig. 2.** Original figures for Fig. 2A and B. **Supplement Fig. 3.** Original figures for Fig. 3A-F. **Supplement Fig. 4.** Original figures for Fig. 4A and C. **Supplement Fig. 5.** Original figures for Fig. 5A and C. **Supplement Fig. 6.** Original figures for Fig. 6A and B. **Supplement Fig. 7.** Original figures for Fig. 7A and B. **Supplement Fig. 8.** Original figures for supplementary Fig. 1A and B.

## Data Availability

All data generated or analyzed during this study are included in this article. Any data supporting the conclusion of this study are available on request.

## Abbreviations

eGSM: The ethanol extract of *Garcinia subelliptica* Merr.
MTT: 3-(4,5-dimethylthiazol-2-yl)-2,5-diphenyltetrazolium bromide
AMPK: AMP-activated protein kinase
mTOR: Mammalian target of rapamycin
ULK1: Unc-51 like autophagy activating kinase
LC3: Microtubule-associated protein 1A/1B-light chain 3
p62/SQSTM1: Sequestosome 1
CRISPR: Clustered regularly interspaced short palindromic repeats
