# Supplementary material for: The ethanol extract of Garcinia subelliptica Merr. induces autophagy
Source: BMC Complement Med Ther. 2021 Nov 10;21:280. doi: 10.1186/s12906-021-03454-4 (PMC8579681; doi:10.1186/s12906-021-03454-4)
Supplement: Supplementary file 1 — Additional file 1: Supplement Fig. 1. A549 (A) or SNU2292 (B) cells were treated with indicated amounts of eGSM for 16 h. Total proteins were extracted, fractionated, and analyzed by western blot with α-p62 antibody. The membranes were stripped and reblotted with α-β-actin antibody. Supplement Fig. 2. Original figures for Fig. 2A and B. Supplement Fig. 3. Original figures for Fig. 3A-F. Supplement Fig. 4. Original figures for Fig. 4A and C. Supplement Fig. 5. Original figures for Fig. 5A and C. Supplement Fig. 6. Original figures for Fig. 6A and B. Supplement Fig. 7. Original figures for Fig. 7A and B. Supplement Fig. 8. Original figures for supplementary Fig. 1A and B. [file 12906_2021_3454_MOESM1_ESM.pptx]

## Slide 1
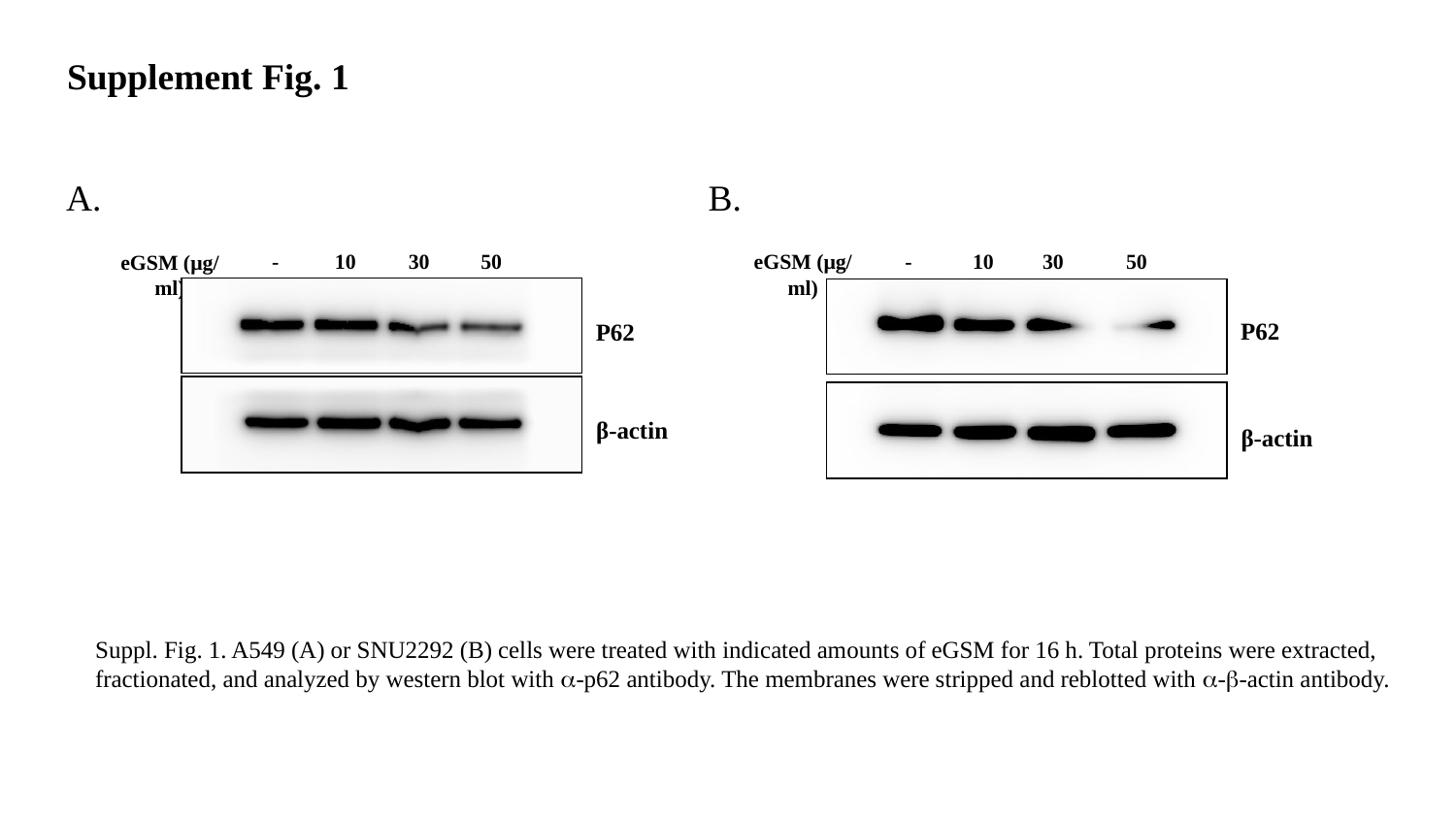

Supplement Fig. 1
A.
B.
-
10
30
50
eGSM (μg/ml)
-
10
30
50
eGSM (μg/ml)
P62
P62
β-actin
β-actin
Suppl. Fig. 1. A549 (A) or SNU2292 (B) cells were treated with indicated amounts of eGSM for 16 h. Total proteins were extracted,
fractionated, and analyzed by western blot with a-p62 antibody. The membranes were stripped and reblotted with a-b-actin antibody.

## Slide 2
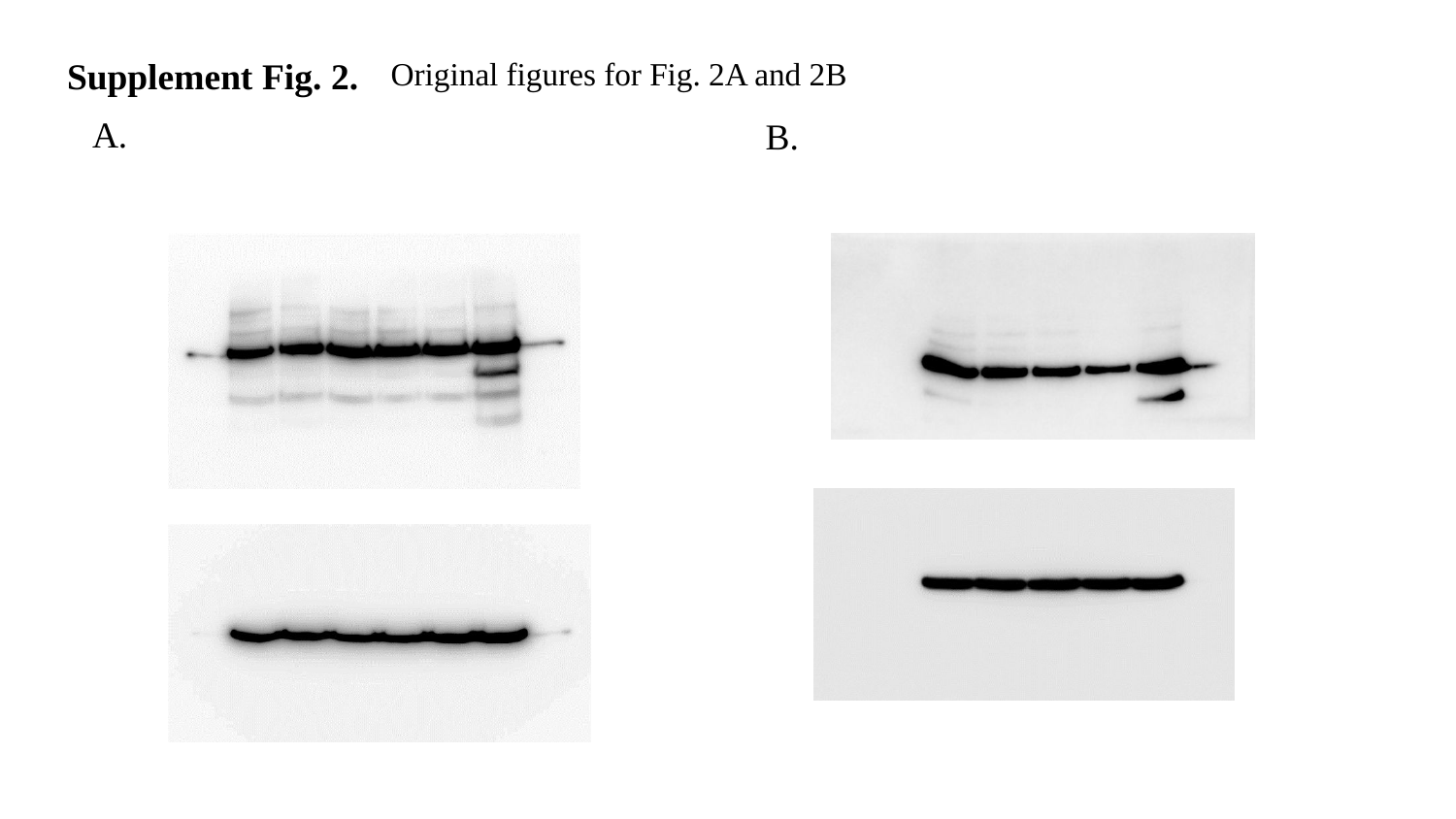

Original figures for Fig. 2A and 2B
Supplement Fig. 2.
A.
B.

## Slide 3
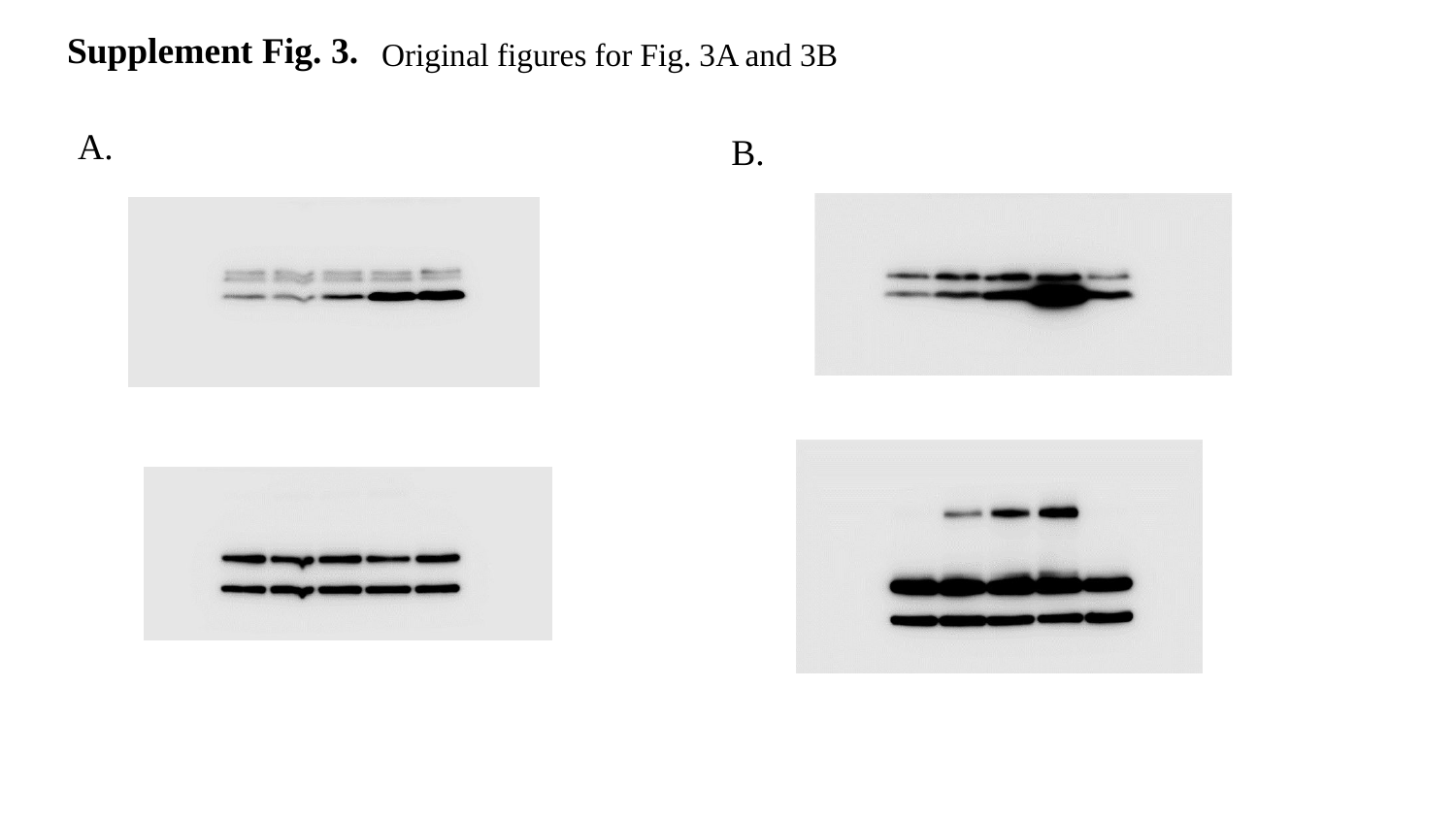

Supplement Fig. 3.
Original figures for Fig. 3A and 3B
A.
B.

## Slide 4
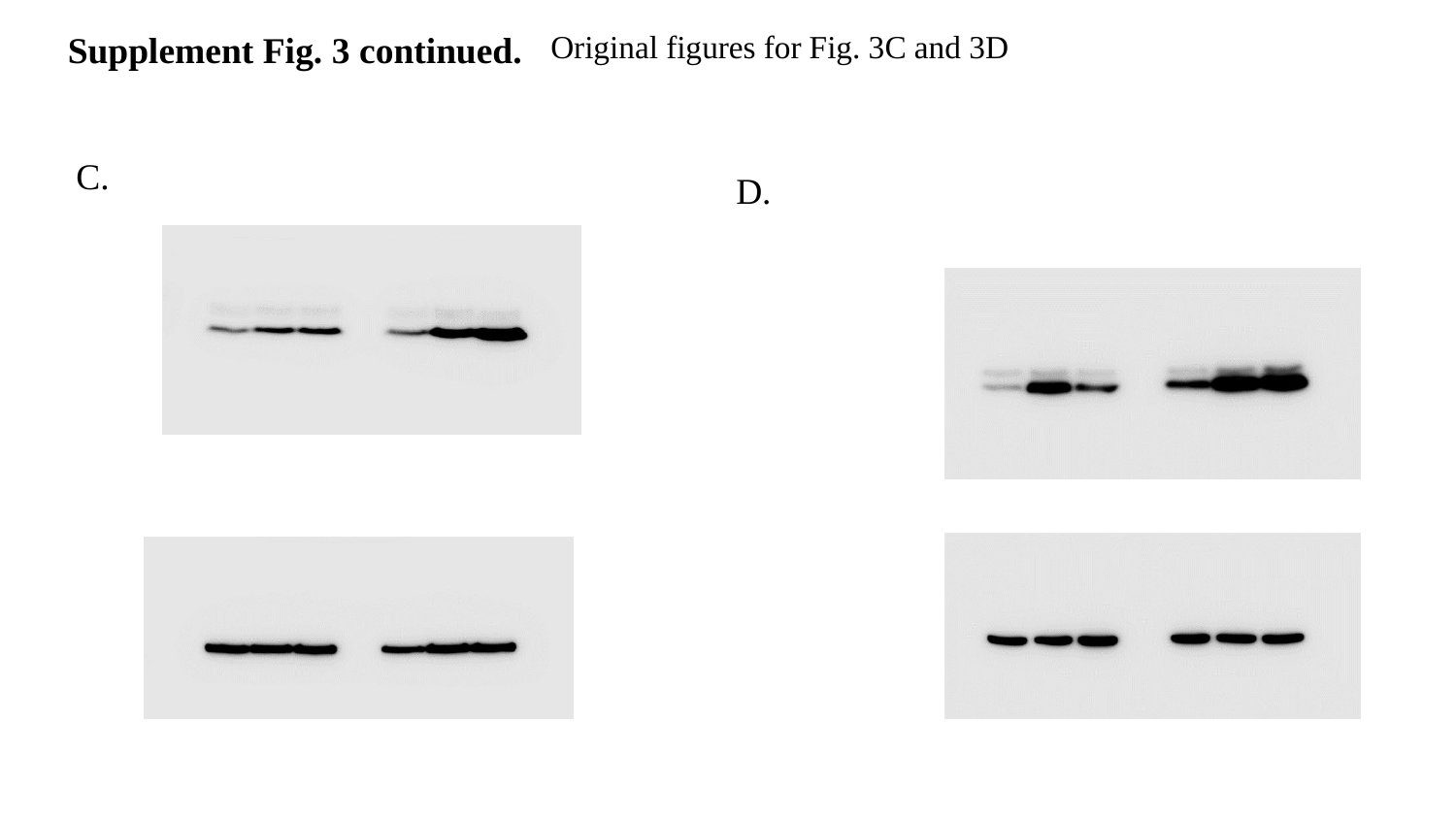

Supplement Fig. 3 continued.
Original figures for Fig. 3C and 3D
C.
D.

## Slide 5
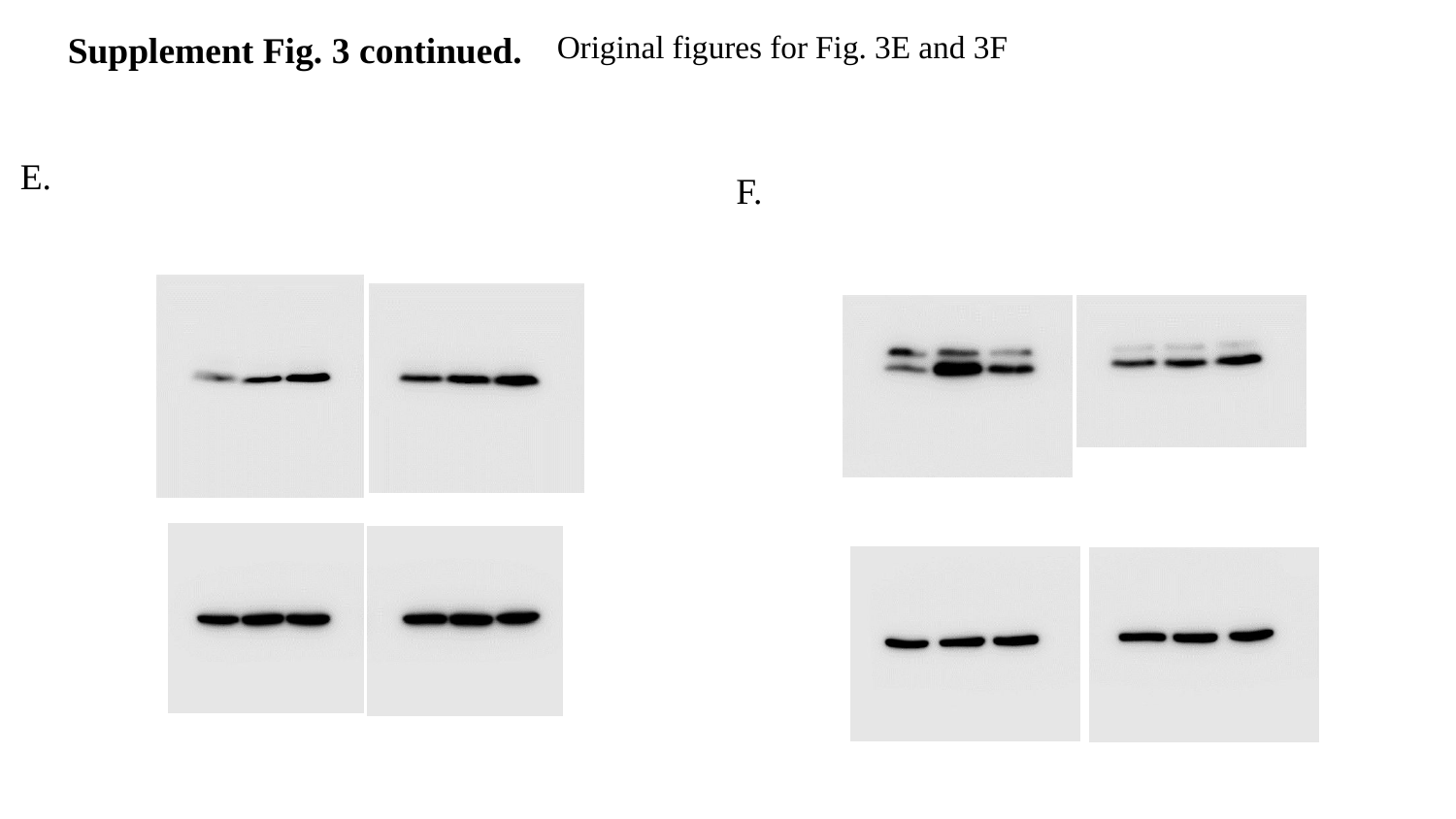

Supplement Fig. 3 continued.
Original figures for Fig. 3E and 3F
E.
F.

## Slide 6
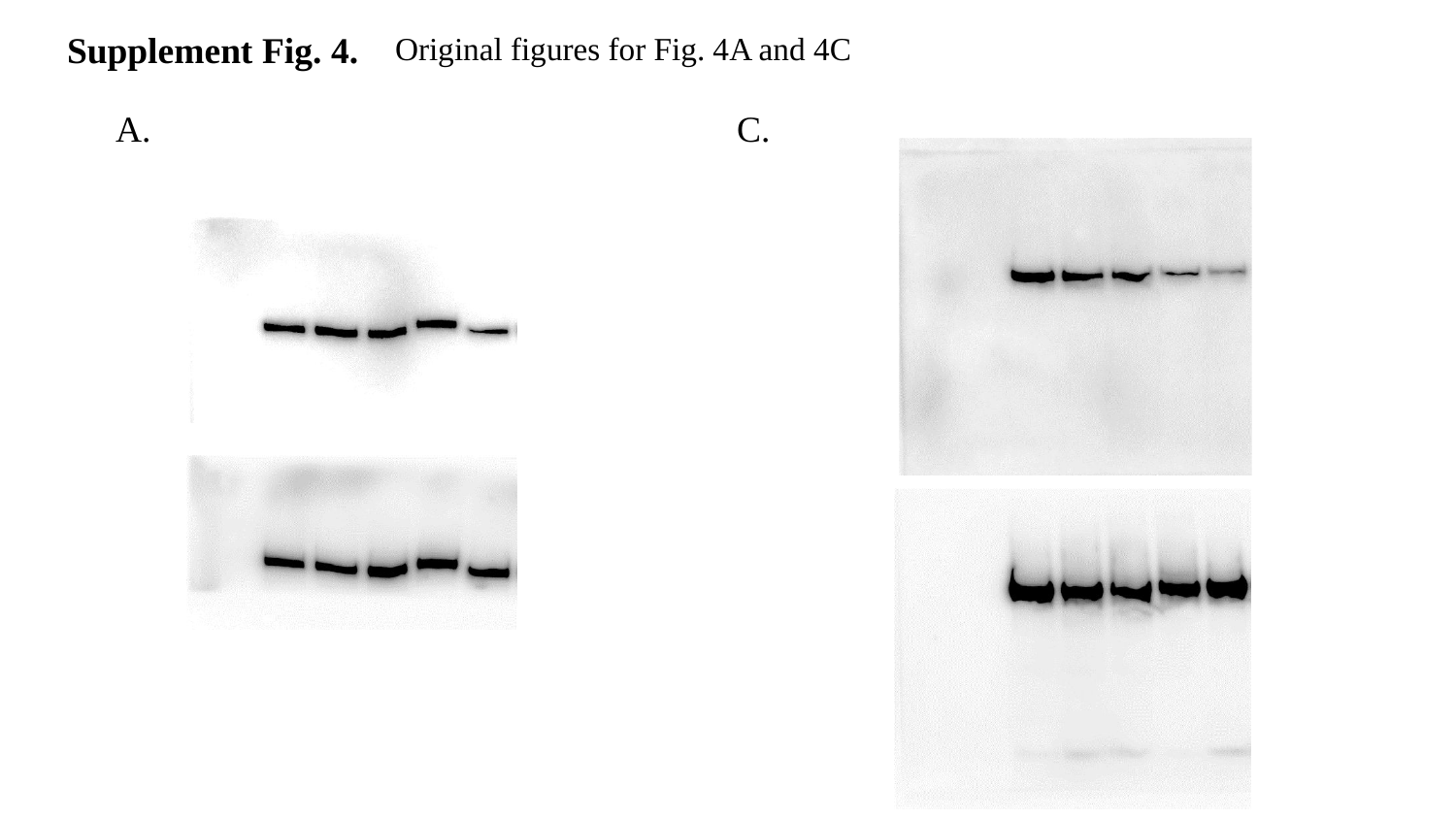

Supplement Fig. 4.
Original figures for Fig. 4A and 4C
A.
C.

## Slide 7
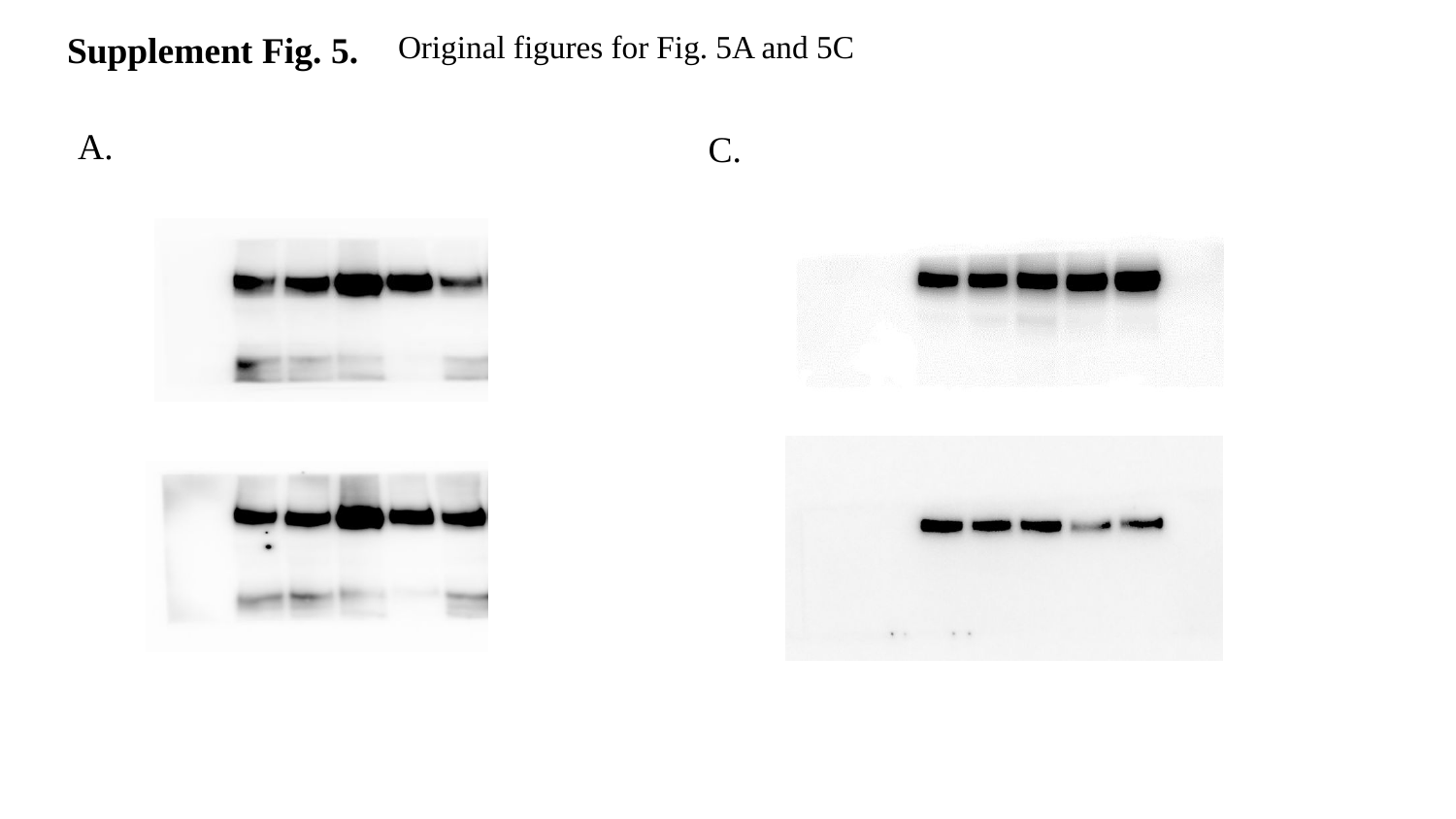

Supplement Fig. 5.
Original figures for Fig. 5A and 5C
A.
C.

## Slide 8
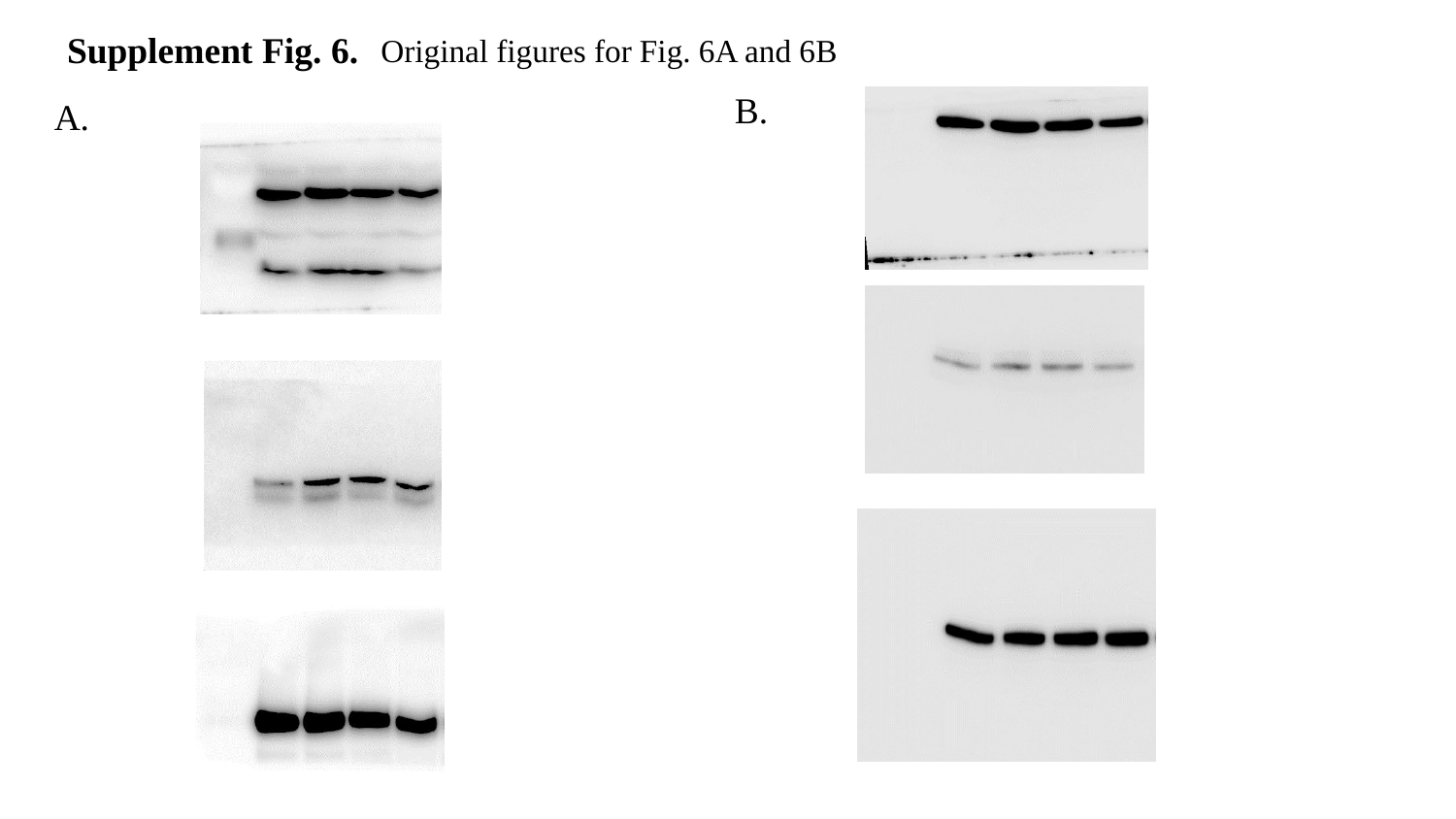

Supplement Fig. 6.
Original figures for Fig. 6A and 6B
B.
A.

## Slide 9
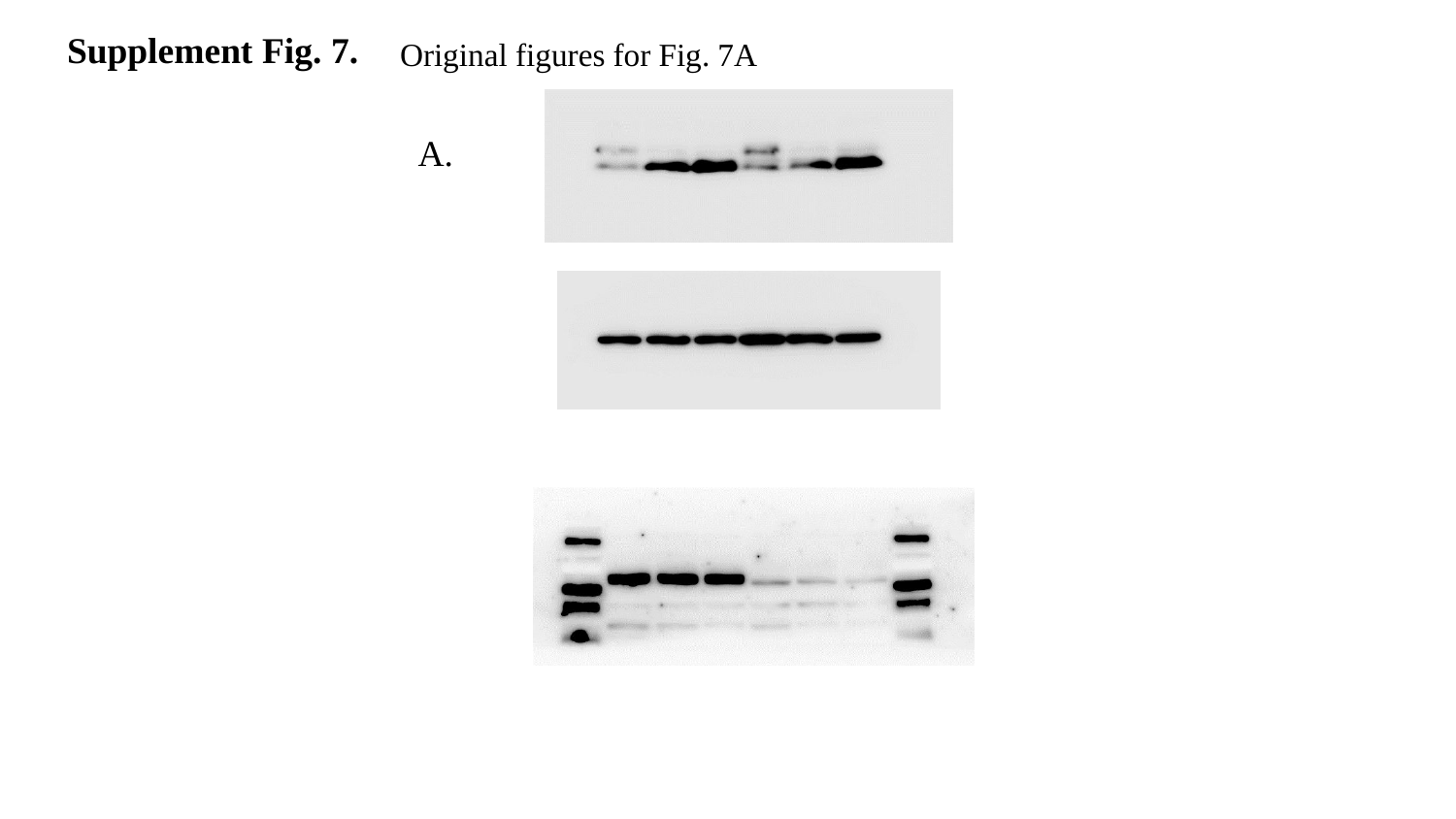

Supplement Fig. 7.
Original figures for Fig. 7A
A.

## Slide 10
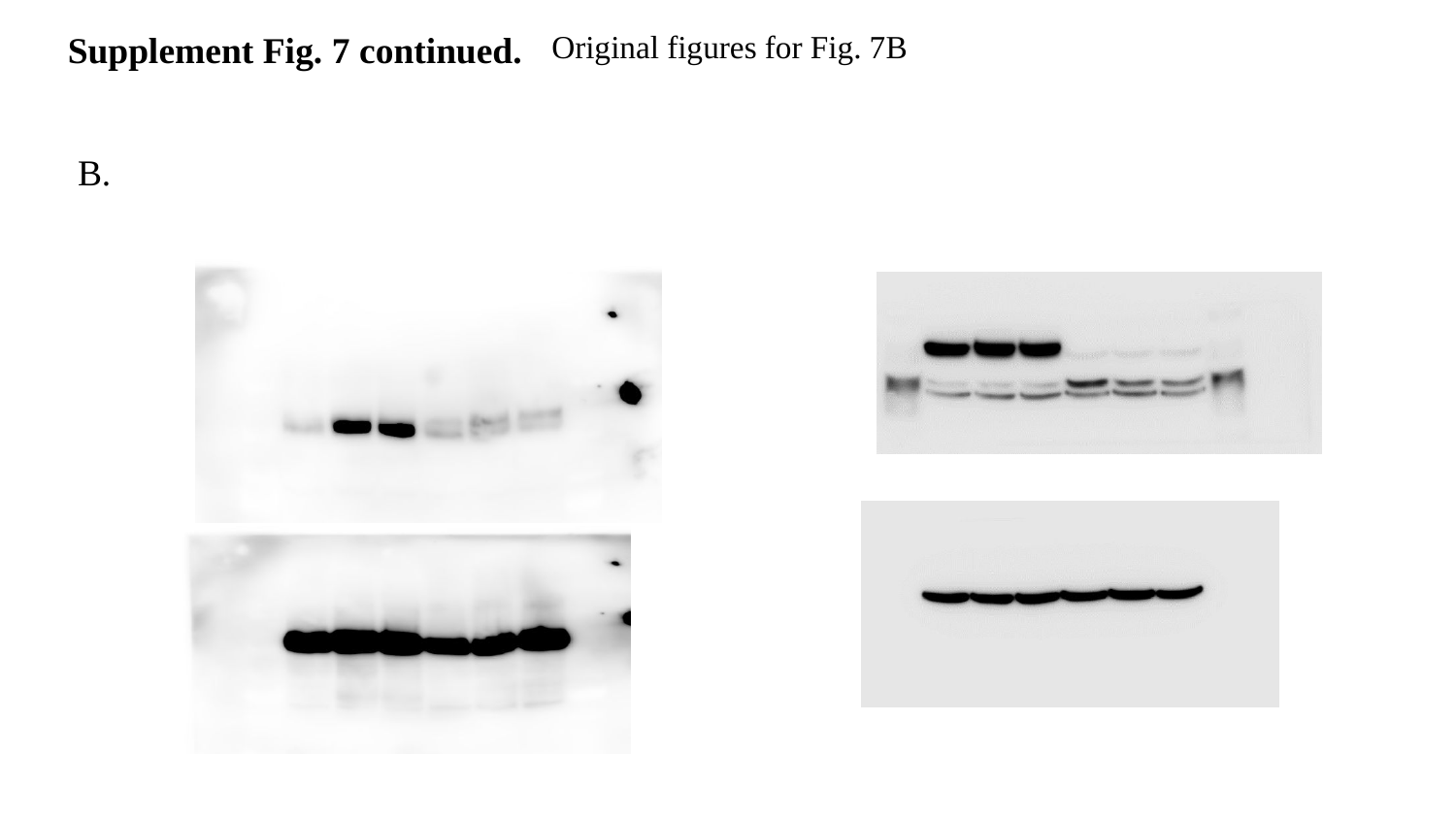

Supplement Fig. 7 continued.
Original figures for Fig. 7B
B.

## Slide 11
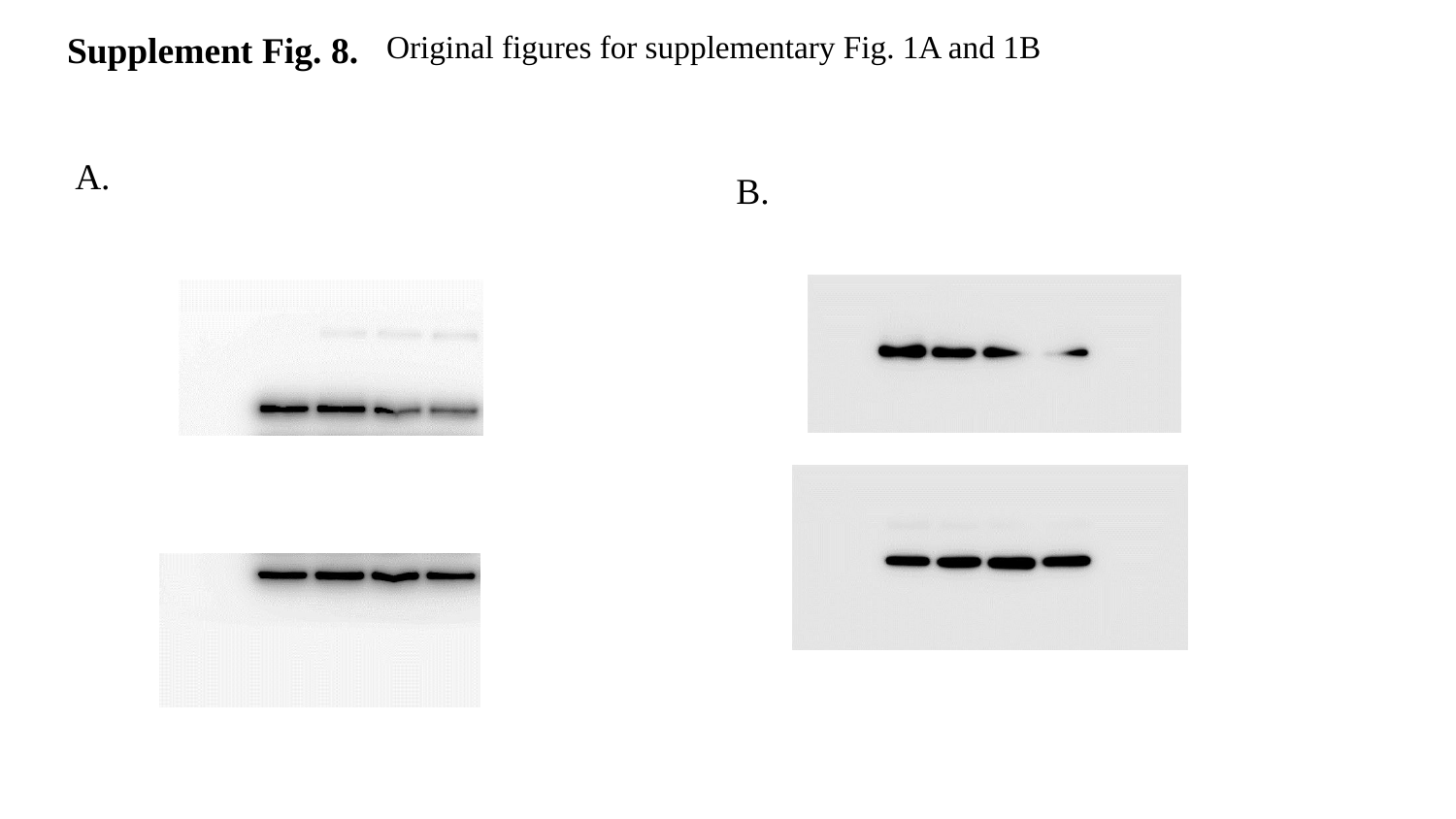

Supplement Fig. 8.
Original figures for supplementary Fig. 1A and 1B
A.
B.
